# Multicriteria optimization of the composition, thermodynamic and strength properties of fly-ash as an additive in metakaolin-based geopolymer composites

**DOI:** 10.1038/s41598-024-61123-1

**Published:** 2024-05-07

**Authors:** Van Su Le, Artem Sharko, Oleksandr Sharko, Dmitry Stepanchikov, Katarzyna Ewa Buczkowska, Petr Louda

**Affiliations:** 1 Institute for Nanomaterials, Advanced Technologies and Innovation, Technical University of Liberec, Bendlova 1409/7, 46001 Liberec, Czech Republic; 2 Kherson State Maritime Academy, Ushakov Ave., Kherson, 73000 Ukraine; 3 Kherson National Technical University, Berislavske Shose, Kherson, 73008 Ukraine; 4 Department of Material Science, Faculty of Mechanical Engineering, Technical University of Liberec, Liberec, Czech Republic; 5 Faculty of Mechanical Engineering, University of Kalisz, Nowy Świet 4, 62-800 Kalisz, Poland

**Keywords:** Markov chains, Intelligent systems, Multicriteria optimization, Geopolymers, Environmental impact, Environmental sciences, Engineering, Materials science

## Abstract

This paper presents the construction of intelligent systems for selecting the optimum concentration of geopolymer matrix components based on ranking optimality criteria. A peculiarity of the methodology is replacing discrete time intervals with a sequence of states. Markov chains represent a synthetic property accumulating heterogeneous factors. The computational basis for the calculations was the digitization of experimental data on the strength properties of fly ashes collected from thermal power plants in the Czech Republic and used as additives in geopolymers. A database and a conceptual model of priority ranking have been developed, that are suitable for determining the structure of relations of the main factors. Computational results are presented by studying geopolymer matrix structure formation kinetics under changing component concentrations in real- time. Multicriteria optimization results for fly-ash as an additive on metakaolin-based geopolymer composites show that the optimal composition of the geopolymer matrix within the selected variation range includes 100 g metakaolin, 90 g potassium activator, 8 g silica fume, 2 g basalt fibers and 50 g fly ash by ratio weight. This ratio gives the best mechanical, thermal, and technological properties.

## Introduction

As high-strength materials, geopolymers are increasingly recognized for their competitive properties with Portland cement, the predominant construction material^1,2^. A critical concern is the substantial environmental impact of Portland cement production, releasing 0.8 tons of CO_2_ into the atmosphere per ton of cement produced, with global cement plants emitting a staggering 1.5 billion tons of CO_2_ annually^3–5^. Geopolymers offer a promising solution by utilizing secondary raw materials such as blast furnace slag and fly ash from thermal power plants, thereby demonstrating eco-friendliness^6–8^. High belite cement^9^ and supersulfated cement^10^ are known for their reduced carbon footprint compared to traditional Portland cement, while geopolymers are also considered low-carbon alternatives. By comparing these different types of low-carbon cement, researchers and engineers can evaluate their performance, environmental impact, and suitability for various applications, helping to advance sustainable construction practices.

Worldwide, intensive research is dedicated to identifying the optimal formulation of geopolymer mixtures that meet specific thermal and mechanical property requirements^11–13^. In such weakly structured systems, numerous factors interconnect, forming a complex web of relationships influenced by both internal and external conditions. However, multiparameter optimization poses significant computational challenges, given the unknown analytical expressions for their boundary values in most practical cases^14^.

The stochastic process of determining the ideal geopolymer composition, guided primarily by expert input without rigorous mathematical processing, involves a series of random experimental measurements governed by optimality criteria. The choice of optimal composition relies heavily on intuitive expert judgment. This process takes on a Markovian character as subsequent decisions become independent of previous ones. The initial probability distribution defines the current state of the experimental input information. Moreover, the selection of the initial step in constructing an intelligent system for optimal geopolymer composition relies on evaluating and adjusting the probabilities associated with key physical and mechanical characteristics, leading to a shift in the reference point. This process continues iteratively, creating a Markov chain with a unique stationary distribution of states after a certain number of transitions^15^.

Some research has focused on activators for geopolymers derived from biomass ash waste, reducing environmental burdens^16^, while other studies have analyzed the suitability of geopolymer composites for withstanding alternating loads^17,18^. Additionally, modeling geopolymers based on pore size distribution and considering particle aggregation into clusters for nanostructure nucleation has been explored^19^. Water sorption in geopolymers has been studied through simulation using the Monte Carlo method and Markov chains^20^. Innovations extend to creating intelligent systems for civil infrastructure repairs, maintenance optimization, and evaluating the optimum content of calcium-rich fly ash in metakaolin-based geopolymers^21–23^.

Furthermore, criteria for calculating the durability of geopolymer bridges reinforced with carbon fiber have been developed^24–26^. The selection criteria for construction materials based on fly ash for optimizing geopolymer compositions have been delineated as part of broader efforts to reduce greenhouse gas emissions^27,28^.

This work's uniqueness lies in its quantitative analysis of alternating loads on a large scale and the associated challenges of choosing optimal solutions when considering multiple optimality criteria. The practical application of Markov chains extends to economic development prediction^29–33^, monitoring lymphatic drainage systems^32^, and studying the COVID-19 pandemic^34^. Additionally, information-entropy models underpin management decisions amidst uncertainty and risk^31^.

In recent studies, the mechanical properties of foamed geopolymers at high temperatures have been assessed, along with the optimization of fly ash parameters in geopolymers^16,18^. Notably, innovative systems have been proposed for studying organizational and technical objects autonomously^21^, modeling technological development^23^, and employing probabilistic models for optimal system trajectory estimation^22,24^.

Given that practical problems often involve multiple criteria for selecting optimal alternatives, the emerging theme in these studies is the use of multicriteria optimization methods to guide decision-making. This approach aims to balance evaluations of different criteria, fostering the transition from subjective to objective optimal solutions. In practice, determining the ideal geopolymer composition involves a process of random expert input, often lacking rigorous mathematical processing, and therefore introducing elements of randomness. Consequently, achieving the desired outcome can be highly dependent on expert qualifications. The primary objective worldwide remains the quest for the optimal formulation of geopolymer mixtures that satisfy specific thermal and mechanical property requirements. This work's focus is squarely on the precision of optimization. The proposed model introduces an innovative approach by integrating multicriteria optimization and Markov chains. The role of Markov chains in the model is to establish the weight coefficients for criteria combinations, enhancing the accuracy and reliability of assessments for geopolymer formulation optimization. The central task of this study was to identify an optimal geopolymer composition that maximizes compressive strength, bending strength, impact toughness, and thermal conductivity while minimizing density and thermal conductivity. In contrast to conventional approaches, this research avoids generalizing or specifying geopolymer composition solely by empirically varying physical and mechanical properties. It tackles the challenge of obtaining an optimal geopolymer formulation while accounting for a multitude of varying properties.

The process of determining optimal geopolymer compositions, observed in these studies, involves a series of randomly selected experimental measurements. In addition to intuition, the selection process retains elements of randomness due to the expertise of the individuals involved. This paper suggests incorporating Markov chains and multicriteria optimization as a more structured and systematic approach to address this limitation. As a result of the model's comprehensive use of multicriteria optimization and Markov chains, the weighting coefficients for criteria combinations are determined by Markov chains. Combining physical and mechanical properties improves the accuracy and reliability of assessments in geopolymer formulation optimization. In addition to examining the physical properties of various geopolymer compositions, this study also aimed to develop a methodology for determining the optimal geopolymer composition based on diverse prioritizations of optimization parameters. To achieve this, samples covering a broad range of physical and mechanical properties were prepared, allowing a deeper understanding of the interrelationships within geopolymer compositions. Under different conditions, this approach allows the prediction of various states of geopolymers, providing valuable insights and practical recommendations.

## Materials, methods, technology, and equipment

The materials employed in this study encompass metakaolin, an activator, carbon fiber, silica fume, and various types of fly ash. The inorganic two-component aluminosilicate binder, commercially designated as Bausik LK and produced by České lupkové závody, a.s. in the Czech Republic, is a metakaolin-based material with a density (ρ = 1220 kg/m^3^) and a chemical composition consisting of 40.10 wt.% Al_2_O_3_, 54.10 wt.% SiO_2_, 0.80 wt.% K_2_O, 1.10 wt.% Fe_2_O_3_, 1.80 wt.% TiO_2_, 0.18 wt.% MgO, 0.13 wt.% CaO, 2.20 wt.% LOI. The grain size distribution is characterized by D50 = 3 μm and D90 = 10 μm. Activated by an aqueous alkaline activator, this binder is renowned for its commendable adhesion, chemical resistance, and ability to withstand extreme temperatures. Typically, a mixing ratio of 5 parts metakaolin to 4 parts activator is employed. Additionally, silica fume sourced from Kema Morava rehabilitation center a.s. in the Republic of Slovenia is incorporated into the mortar. This silica fume exhibits a density (ρ = 350 kg/m^3^) and a chemical composition comprising 90 wt.% SiO_2_, 1 wt.% Al_2_O_3_, 0.8 wt.% CaO, 1.5 wt.% MgO, 0.5 wt.% Na_2_O. The average grain size is 100 μm. Recycled carbon fibers with a density (ρ = 1800 kg/m^3^) and a chemical composition of > 95 wt.% C, with an average length of 6 mm, are employed as reinforcing fibers. These chopped fibers are particularly well-suited for the production of dry and molding mortars. The geopolymer production process involves the incorporation of fly ashes labelled as FA1-7 (refer to Table 1 and 2) obtained from thermal power plants in the Czech Republic. These fly ashes, characterized by densities of 625.89, 645.53, 669.08, 667.89, 702.92, 692.05, and 623.23 kg/m^3^, respectively, are utilized in the production process. The chemical compositions of the raw materials were analyzed using X-ray fluorescence (BRUKER S8 Tiger instrument, BRUKER, Karlsruhe, Germany) and scanning electron microscopy (SEM Carl Zeiss Ultra Plus). The findings are presented in Table 1. These materials have been comprehensively elucidated in the research paper titled "Multicriteria Assessment for Determining the Optimal Proportion of Calcium-Rich Fly Ash in Metakaolin-Based Geopolymers".

**Table 1:** XRD analysis detects crystalline phases in fly ash types.

Fly ash	Crystalline phase—chemical formula						
	CaCO_3_	SiO_2_	K_2_Ca(SO_4_)_2 *_ H_2_O	MgCO_3_	Al_2_O_3_	K_2_SO_4_	Al_2_O_3_
FA1	35.2	37.1	27.7	–	–	–	–
FA2	42.7	55.8	–	0.9	0.5	–	–
FA3	35.2	37.1	–	–	–	27.7	–
FA4	34.0	35.2	–	–	–	30.8	–
FA5	39.7	39.0	–	–	–	21.3	–
FA6	39.9	38.2	–	–	–	21.9	–
FA7	31,3	29.7	–	–	–	38.4	0.6

**Table 2:** The ratios and types of the constituent materials used in all mixtures.

No. samples	Metakaolin (g)	Activator (g)	Fly ash (g)	Carbon fiber (g)	Silica fume (g)	No. fly ash
1	100	90	100	2	8	FA1
2			75			
3			50			
4			100			FA2
5			75			
6			50			
7			100			FA3
8			75			
9			50			
10			100			FA4
11			75			
12			50			
13			100			FA5
14			75			
15			50			
16			100			FA6
17			75			
18			50			
19			100			FA7
20			75			
21			50			

The preparation of the geopolymer mix entailed a sequential process. Initially, cement-based metakaolin and an alkaline potassium activator were combined in a proportion of 5:4, as prescribed by the manufacturer, and subjected to thorough mixing for three minutes. Subsequently, the inclusion of silica fume, fly ash, and fibers into the geopolymer mortar followed, with the mixing process extended to a duration of five minutes. These procedures collectively influenced the characteristics of the freshly prepared geomortar. The resultant mixture was then cast into molds, and a protective layer of polyethylene film was applied to mitigate shrinkage during the curing process. Subsequently, the specimen was allowed to undergo curing at ambient room temperature for 28 days, in preparation for subsequent testing and analysis. The ratios and types of the constituent materials used in all mixtures are presented in Table 2.

The input information for the physical and mechanical properties of different concentrations of fly ash in the composition of geopolymer mixtures included tests for bending, compression, impact strength, and measurement of density and thermal conductivity.

**Figure 1:**
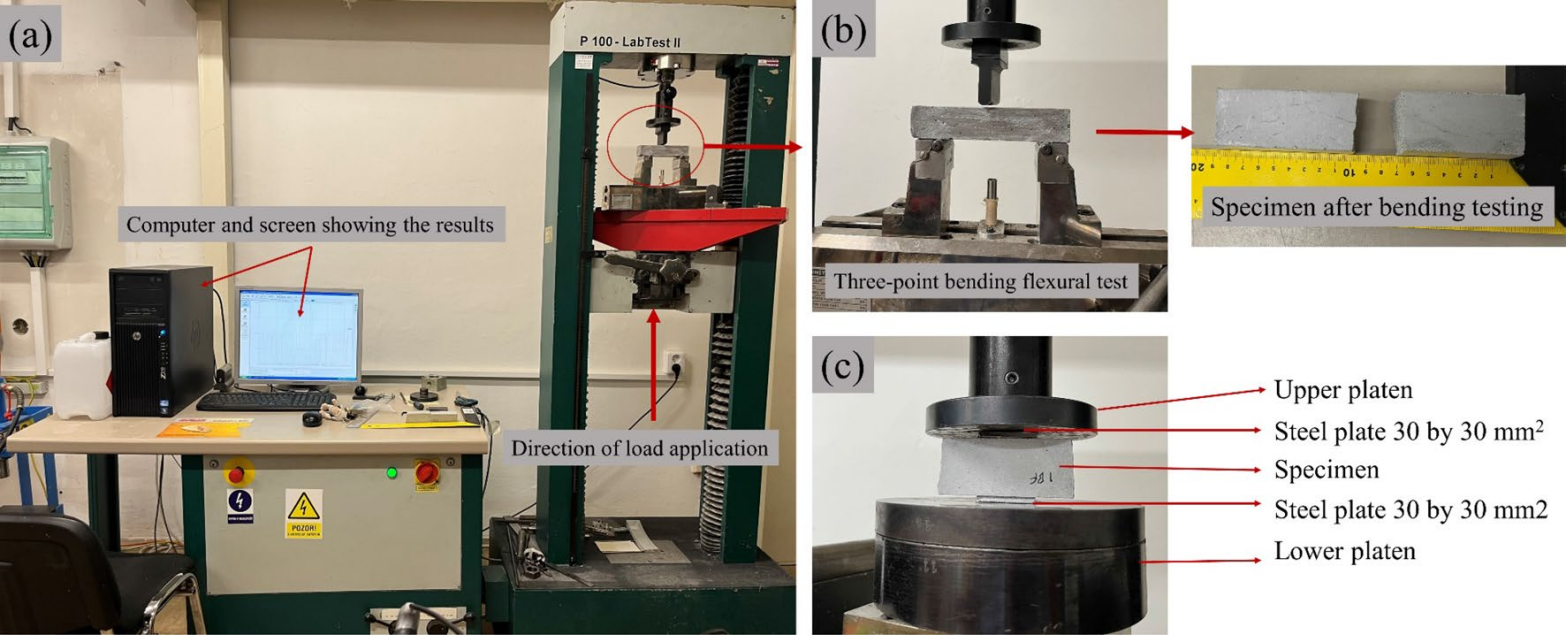
Stress testing setup: (**a**) Instron model 4202 testing machine, (**b**) bending testing setup, (**c**) compression testing setup.

**Figure 2:**
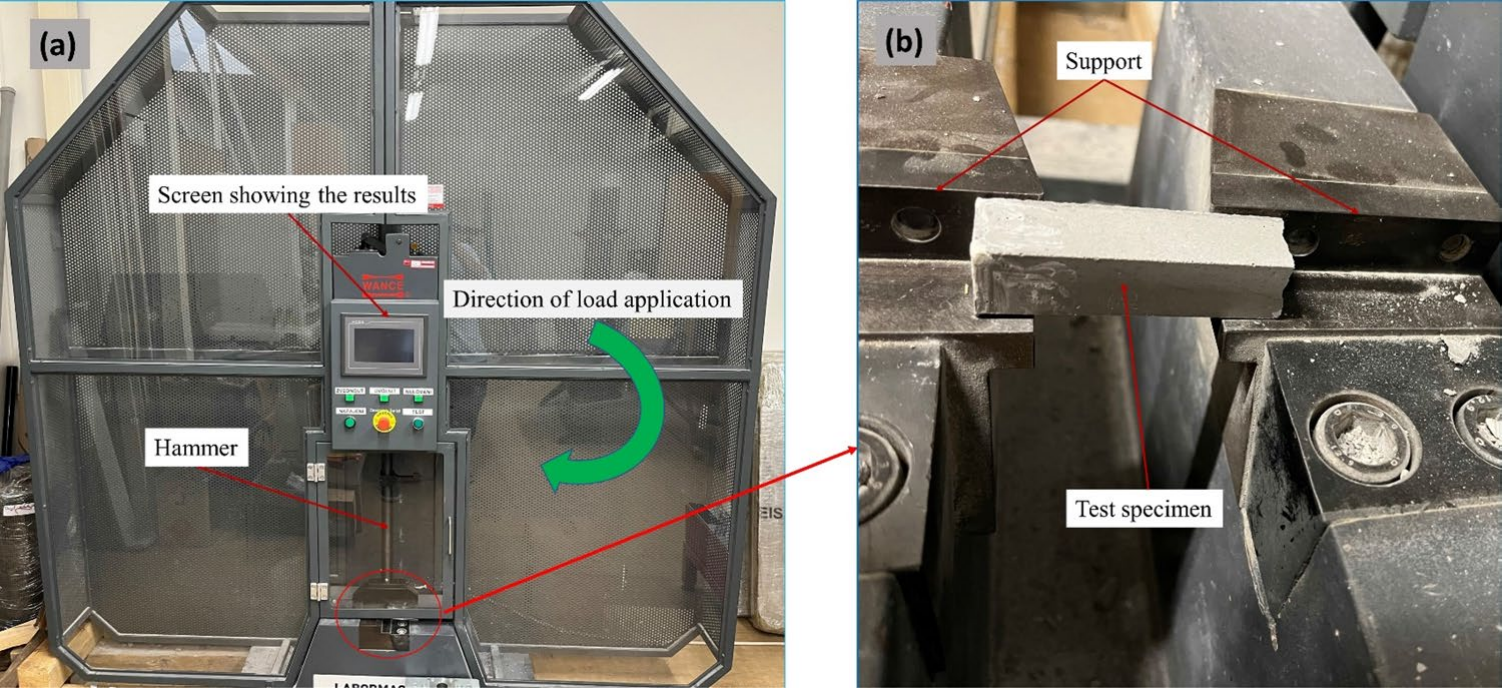
Charpy impact test setup: (**a**) universal testing machine, (**b**) specimen testing setup.

The apparent density was estimated by dividing the mass of the sample by its apparent volume. The samples have dimensions of 30 by 30 by 150 mm^3^. Bending and compression tests were carried out on an Instron (Model 4202) Universal Testing Machine with a 10 kN load cell traverse speed of 2.5 mm/min according to the UNI EN 1015–11:2019 standard^35^. The compressive strength tests were carried out on the same specimens as the bending tests, and their geometric dimensions were 30 by 30 by 30 mm^3^ (Fig. 1). The Charpy impact strength was determined using a PIT-C Series Pendulum Impact Testing Machine on samples measuring 20 by 19 by 60 mm^3^ by the ISO 148–1:2016 test method^36^ (Fig. 2). The setup for stress tests is depicted in Figs. 1 and 2. Using test samples with dimensions of 300 by 300 by 30 mm^3^, the thermal conductivity was investigated using the NETZSCH HFM 446 instrument.

The determination of priorities and preferences in multicriteria optimization is based on the fact that there exists a function U, whose domain of definition is determined by the preference criteria A and B, with A being preferable to B.

1 $$U\left(A\right)>U\left(B\right)\iff A>B,$$

If A = B, then there is a state of indifference

2 $$U\left(A\right)=U\left(B\right)\iff A=B.$$

At the same time, the presented calculation scheme provides only the ranking of criteria, not their numerical values. The simultaneous optimization of two or more conflicting target functions in a given domain of definition is multicriteria. A multiattribute preference function with three target functions can be represented as follows.

3 $$U={k}_{1}{U}_{1}+{k}_{2}{U}_{2}+{k}_{3}{U}_{3}+{k}_{1}{k}_{2}{U}_{1}{U}_{2}+{k}_{2}{k}_{3}{U}_{2}{U}_{3}+{k}_{3}{k}_{1}{U}_{3}{U}_{1}+{k}_{1}{k}_{2}{k}_{3}{U}_{1}{U}_{2}{U}_{3},$$

In Eq. (3) there are three target functions and three constants, i.e. we have a definition of the best priority on the admissible set of optimality criteria.

Markov chains are a method of modeling random events that represent a discrete sequence of phases, each of which is located at discrete points in the state space.

Applied to the research problem, they are a tool of the theory of random processes consisting of a sequence of states. This is interpreted as the sum of several probabilities of some event K, depending on the occurrence of state I, where event K = m + 1, I = m, i.e. event K differs from I by 1.

4 $$\sum_{k=1}^{r}{p}_{ik}=\sum_{k=1}^{r}P({X}_{m+1}=k|{X}_{m}=i),$$

where *r* is the matrix rank.

The probability distribution in a Markov chain depends only on transitions from the current state to the next state. Therefore, a Markov chain is represented as a sequence of states n ∈ N. Mathematically it looks like this.

5 $$X={({X}_{n})}_{n\in N}=\left({X}_{0},{X}_{1},{X}_{2},\dots \right),$$

where at each moment the process takes such values in the discrete set E that

6 $${X}_{n}\in E \,\,\,\,\,\,\,{\forall }_{n}\in N.$$

where ${\forall }$ is the quantum of generality:

Let us denote all possible states of the system as S_1_, S_2_, … S_n_, then

7 $$P\left({X}_{n+1}={s}_{n+1}|{X}_{n}={s}_{n},{X}_{n-1}={s}_{n-1},{X}_{n-2}={s}_{n-2},\dots \right)=P\left({X}_{n+1}={s}_{n+1}|{X}_{n}={s}_{n}\right).$$

The transition probabilities from one state to another are represented as square matrices.

8 $${P}_{ij}\left(n\right)=P\left({x}_{n+1}=j|{x}_{n}=i\right),$$

9 $$P = \begin{array}{*{20}c} {} & {\begin{array}{*{20}c} {s_{1} } & {s_{2} } & {...} & {s_{n} } \\ \end{array} } \\ {\begin{array}{*{20}c} {s_{1} } \\ {s_{2} } \\ . \\ {s_{n} } \\ \end{array} } & {\left[ {\begin{array}{*{20}c} {p_{11} } & {p_{12} } & {...} & {p_{1n} } \\ {p_{21} } & {p_{22} } & {...} & {p_{2n} } \\ . & . & {...} & . \\ {p_{n1} } & {p_{n2} } & {...} & {p_{nn} } \\ \end{array} } \right]} \\ \end{array} ,$$

When passing from one row to the next in the transition probability matrix, an induced stationary distribution over the rows appears. Let us denote by Pi0(0) the probability of starting the process from the state Si. Pij0(0) is the probability that as a result of the process, the state will change from Si to Sj. Pij is the probability that the result of the calculation will be Si

10 $${P}_{ij}\left(0\right)={P}_{1}\left(0\right)\cdot {P}_{1i}+{P}_{2}\left(0\right)\cdot {P}_{2i}+{P}_{3}\left(0\right)\cdot {P}_{3i}+...+{P}_{n}\left(0\right)\cdot {P}_{ni}.$$

Multiplying the line describing the probability distribution at a certain stage of determining the composition of geopolymer matrices corresponding to optimal mechanical properties by the transition probability matrix, we obtain the probability distribution at the next stage.

Thus, Markov chains combine a priori assumptions and experimental data to obtain the a posteriori distribution of the parameter of interest.

When establishing criteria for assessing the strength of geopolymers, it is necessary to select such benchmarks that cover the conditions of realization of the objective as much as possible. For the successful selection of alternatives for geopolymer formulation, it is necessary that all variants of work are compatible with each other, i.e. they could be comparable in terms of the factors of geopolymer structure. To compare the alternatives, a score is assigned to each of them and the final evaluation is performed by prioritizing them on a ranking scale.

The synthesis of ideas on multicriteria optimization of geopolymer composition is based on the use of efficiency algorithms included in the definition of the evaluation functional F = {f_*jk*_}, and computations are performed to find the optimal solution x_0_ ∈ X according to the chosen criterion.11

In expanded form, this procedure will be characterized by a matrix, elements f_*jk*_ which are quantitative estimates of the quantity *x*_*k*_ ∈ X corresponding to experimental values *y*_*k*_ ∈ Y.

An evaluation functional *F* has positive ingredient *F* + if the decision is based on the condition that xmax *k* ∈ *X*{*f*_*jk*_}. In this case.

12 $$F={F}^{+}=\left\{{{f}_{jk}}^{+}\right\}.$$

The negative ingredient is based on the condition of achieving a *x*_*mink*_ ∈ *X*{*f*_*jk*_}. In this case.

13 $$F={F}^{-}=\left\{{{f}_{jk}}^{-}\right\}.$$

The value of generalized estimates characterizes the degree of approximation to the goal.

Both the averaging function and the maximin function can be used as generalizing functions.

Optimization of geopolymer mixture composition consists of finding a solution, at which the values of target functions would be acceptable for the task.

Decision-making criteria for selecting the optimal formulation of geopolymer mixtures to ensure maximum mechanical properties can be considered as preference operations on the set of alternatives, providing an interactive solution to optimization problems. The optimization criteria are not extreme, but some compromise values correspond to the main objective of the optimization.

The range of variation of the resulting characteristics of strength and physical–mechanical properties is determined by setting the extrema of the boundaries of the target values of the criteria.

In simple terms, the optimization of a geopolymer mixture aims to improve its physical properties, such as density and strength (flexural, compressive, and impact), by using a scalar function that gives a linear ranking of the results. This is achieved by reducing the density and converting vector estimates to scalar ones, as the target orientations and dimensions are different. The scalar functions based on the extreme values of the mixture.

The procedure of determining the optimal geopolymer composition based on the analysis of physical–mechanical and strength parameters begins with the construction of the efficiency matrix, which looks as follows^23^.

14 $$R = \left( {\begin{array}{*{20}c} {} & {\begin{array}{*{20}c} {\Pi_{1} \;\;} & {\Pi_{2} \;} & {...\;} & {\Pi_{j} } \\ \end{array} } \\ {\begin{array}{*{20}c} {q_{1} } \\ {q_{2} } \\ {...} \\ {q_{i} } \\ \end{array} } & {\begin{array}{*{20}c} {\delta y_{11} } & {\delta y_{12} } & {...} & {\delta y_{1j} } \\ {\delta y_{21} } & {\delta y_{22} } & {...} & {\delta y_{2j} } \\ {...} & {...} & {...} & {...} \\ {\delta y_{i1} } & {\delta y_{i2} } & {...} & {\delta y_{ij} } \\ \end{array} } \\ \end{array} } \right),$$

where q1…qi is the fly ash content in the geopolymer mixture, Π1…Πj is the physical–mechanical and strength parameters of geopolymer, i is the line number, j is the column number.

The relative deviation δy_*ij*_ jth attribute from the optimal value is determined as follows.

15 $$\delta y_{ij} = \left\{ {\begin{array}{*{20}c} {\frac{{\left| {y_{ij} - c_{j} } \right|}}{{y_{j,\max } - c_{j} }};y_{ij} > c_{j} } \\ {\frac{{\left| {y_{ij} - c_{j} } \right|}}{{c_{j} - y_{j,\min } }};y_{ij} < c_{j} } \\ \end{array} } \right.$$

As parameters it is necessary to choose the best values of the analyzed parameters from the point of view of the problem to be solved—it can be maximum or minimum from the experimental sample. In such an approach, formula (15) will translate dimensional values into a ratio within the scale (0, 1). However, with such a choice of parameters cj will necessarily be observed coinciding with the value of cj corresponding elements of matrix (Eq. 14), which will lead to δyij = 0. When using additive convolution (Eq. 16), this leads to the dropping of the corresponding attribute from the overall evaluation of the object, and when using multiplicative convolution (Eqs. 17, 18) to its nullification. The obvious way to exclude such situations is to extend the upper (for maximum) or lower (for minimum) limit of each feature c_j_ in the same percentage ratio. Below the maximum (minimum) values of each of the analyzed parameters, c_j_ was increased (decreased) by 1%.

To select the optimal combination of the composition of fly ash components and to ensure maximum strength properties at minimum density and thermal conductivity of the geopolymer mixture, it is necessary to take into account more than one criterion. Therefore, it is necessary to reconcile such multidirectional criteria.

The following generalized multicriteria utility functions were used in the theoretical analysis^23,37^.

Additive convolution:

16 $$y_{a} = \delta y_{i} = \sum\limits_{j = 1}^{n} {\omega_{j} \delta y_{ij} } ,$$

where *ω*_*j*_ is the weight coefficient of the jth attribute.

Stepwise multiplicative convolution:

17 $$y_{ms} = \delta y_{i} = \prod\limits_{j = 1}^{n} {\left( {\delta y_{ij} } \right)^{{\omega_{j} }} } ,$$

Additional multiplicative convolution:

18 $$y_{md} = \delta y_{i} = 1 - \prod\limits_{j = 1}^{n} {\left( {1 - \omega_{j} \delta y_{ij} } \right)} ,$$

The fly ash composition having a minimum value of functions (Eqs. 16–18) is considered to be the best.

Wald criterion (minimum maximum):

19 $$Z_{v} = \mathop {\min }\limits_{i} \mathop {\max }\limits_{j} \delta y_{ij} ,$$

Laplace criterion (minimum minimum):

20 $$Z_{L} = \mathop {\min }\limits_{i} \mathop {\min }\limits_{j} \delta y_{ij} ,$$

Since obtaining the thermal and physical–mechanical properties of geopolymers by changing their formulations does not have a constant time reference, we will use the steps that characterize the successive approximation of the approach states to the achievement of the intended goal as the time. In this case, time is replaced by the step number with a hierarchy of discretization intervals as an argument. This is an undoubted novelty and innovation in solving the problems of the process of constructing an intelligent system of optimality criteria.

The points of innovation in the presented methodology are as follows:

- Replacement of expert evaluation in the selection of multidirectional geopolymer strength orientations by mathematical justification;
- construction of a conceptual model of the selective choice of geopolymer formulation using Markov chains and multicriteria optimization;
- rank-based approach to optimization criteria;
- replacement of time references by discretization intervals;
- scalability and adaptation to loadings through scaling;
- scalarization of vector estimates.

Conditional probabilities of optimality criteria are presented in Table 3.

**Table 3:** Conditional probabilities of optimality criteria.

Current state	Subsequent state				
	Compressive strength	Bending strength	Impact strength	Density	Thermal conductivity
Compressive strength	0.30	0.26	0.18	0.16	0.10
Bending strength	0.33	0.24	0.16	0.14	0.13
impact strength	0.34	0.18	0.14	0.14	0.20
Density	0.31	0.22	0.16	0.13	0.18
Thermal conductivity	0.29	0.24	0.15	0.16	0.16

The construction of Table 3 begins with filling in its first row based on expert judgments and a priori knowledge about the subject area. The formalization of the presented knowledge in the form of Table 3 parameters is implemented using logical inference and evolution of iteration steps.

## Results and discussion

When compiling the matrix of transition probabilities, the values of parameters of the conditional probability of current and subsequent states of strength properties of geopolymers when changing the composition and technology of their manufacture are determined from expert assessments by studying the basic phenomenological assumptions reflected in the mechanisms of structure change through relative magnitudes.

Compressive strength, which determines the maximum weight a building material can bear, is manifested in changes in properties such as deformability under increasing pressure, elasticity level depending on the number of pores in the mortar, spreading or creep, shrinkage, swelling, frost resistance, and resistance to chemical influences.

The bending strength depends on the irregularity of shrinkage, modes, and types of curing, and conditions of processes in air or water.

Impact toughness is determined by the work at impact, which is manifested by the initial height of the pendulum before impact and the height of the pendulum after impact. This characteristic is related to compressive and bending strengths through the mechanism of structure formation. However, the quantitative parameters of such a relationship require certain conditions to be met and are therefore expressed in our work through probabilistic parameters.

A separate parameter affecting the complex strength characteristics is density, which determines the structure of the geopolymer mixture. The interaction between the density and strength characteristics of geopolymer concrete is weakly structured. It is pro.

Thermal conductivity as a thermophysical characteristic of geopolymers, in addition to its main purpose, is associated with correlations with the linear stretching coefficient and direction of stretching, linear shrinkage, impact toughness, and thermal resistance. Functional relationships between these events have not been established due to a lack of information or the complexity of these relationships. The structure of geopolymers has gas-filled pores that open at high temperatures.

The presented aspects of the strength and thermophysical properties of geopolymers and their interrelationships through the mechanisms of structure formation were taken as a basis for the construction of the transition probability matrix.

Difficulties arising in the ranking of multicriteria optimization criteria show that experience and intuition are not always the keys to a successful solution.

A useful tool for these purposes is simulation modeling, which allows for the purposeful generation of decision-making options, meaningful selection of structures, and programming of points of interest.

The priority of the arrangement of conditional probabilities of strength properties was determined based on experimental assessments of the role of this parameter in the mechanism of geopolymer structure formation, taking into account that the sum of conditional probabilities in the row of the matrix is equal to one. The columns of the conditional probability matrix, characterize the number of iterations and transitions of the system during its evolutionary transformations and should not be summed.

In this case, it is envisaged to set the weighting coefficients in the form of the probability distribution for all admissible values of Markov chains. This approach allows us to take into account the a priori distribution overall output parameters and to calculate the posterior distribution by the generated assumptions.

Each state of the parameters characterizing the information situation of determining the thermophysical and physical–mechanical properties of geopolymers at a given formulation of their production is assigned a certain probability recorded in the form of a line of the matrix of states. The matrix of intensities or transitions of the system describes the wandering of the system through its states. In the process of analyzing the state matrix, all possible states of the parameters are renumbered with their probabilities, i.e., we deal with a set of probability values between iterations. These iterations are performed for different geopolymer strength parameters.

The initial state vector in Table 3 can be written in the form:

21 $$P\left(0\right)=\left[0.300, 0.260, 0.180, 0.160, 0.100\right].$$

The transition probability matrix has the following form:

22 $$T=\left[\begin{array}{ccccc}0.300& 0.260& 0.180& 0.160& 0.100\\ 0.330& 0.240& 0.160& 0.140& 0.130\\ 0.340& 0.180& 0.140& 0.140& 0.200\\ 0.310& 0.220& 0.160& 0.130& 0.180\\ 0.290& 0.240& 0.150& 0.160& 0.160\end{array}\right].$$

Multiplying the initial state vector P(0) by the transition probability matrix T, we obtain the probability distribution at the first decision stage P(1). According to the method of calculating Markov chains, this probability will be equal to:

23 $$P\left(1\right)=P\left(0\right)\times \left[\begin{array}{ccccc}0.300& 0.260& 0.180& 0.160& 0.100\\ 0.330& 0.240& 0.160& 0.140& 0.130\\ 0.340& 0.180& 0.140& 0.140& 0.200\\ 0.310& 0.220& 0.160& 0.130& 0.180\\ 0.290& 0.240& 0.150& 0.160& 0.160\end{array}\right]=\left[\mathrm{0.316,0.232,0.161,0.146,0.145}\right].$$

Multiplying the state vector P(1) by the transition probability matrix T, we obtain the probability distribution for the next stage of decision-making P(2). The probability P(2) that, being in state S_1_ of the information system, the decision will move to state S_o_, characterized by parameter ν_2_, is equal to:

24 $$P\left(2\right)=P\left(1\right)\times \left[\begin{array}{ccccc}0.300& 0.260& 0.180& 0.160& 0.100\\ 0.330& 0.240& 0.160& 0.140& 0.130\\ 0.340& 0.180& 0.140& 0.140& 0.200\\ 0.310& 0.220& 0.160& 0.130& 0.180\\ 0.290& 0.240& 0.150& 0.160& 0.160\end{array}\right]=\left[\mathrm{0.313,0.234,0.162,0.148,0.143}\right].$$

Multiplying the state vector P(2), characterized by the parameter ν_2_, by the transition probability matrix T, we obtain the probability distribution of the next decision step P(3).

25 $$P\left(3\right)=P\left(2\right)\times \left[\begin{array}{ccccc}0.300& 0.260& 0.180& 0.160& 0.100\\ 0.330& 0.240& 0.160& 0.140& 0.130\\ 0.340& 0.180& 0.140& 0.140& 0.200\\ 0.310& 0.220& 0.160& 0.130& 0.180\\ 0.290& 0.240& 0.150& 0.160& 0.160\end{array}\right]=\left[\mathrm{0.313,0.233,0.162,0.148,0.144}\right].$$

The transition probability of the system from state S3 to state S4 is determined by multiplying P(3) by the transition probability matrix.

26 $$P\left(4\right)=P\left(3\right)\times \left[\begin{array}{ccccc}0.300& 0.260& 0.180& 0.160& 0.100\\ 0.330& 0.240& 0.160& 0.140& 0.130\\ 0.340& 0.180& 0.140& 0.140& 0.200\\ 0.310& 0.220& 0.160& 0.130& 0.180\\ 0.290& 0.240& 0.150& 0.160& 0.160\end{array}\right]=\left[\mathrm{0.313,0.233,0.162,0.148,0.144}\right].$$

It follows from the theory of Markov chains that the shorter the cycle length, the more accurate analyses can be performed. As seen, starting from the third step, the probabilities came to a stationary state in which the probability values correspond to the weight coefficients of the parameters for optimizing the strength properties:

- Compressive strength 0.313
- Bending strength 0.234
- Impact strength 0.162
- Density 0.147
- Thermal conductivity 0.144

The analysis of step-by-step calculations of conditional probabilities shows that the ranking should start with the compressive strength characteristics, the next strength criterion will be bending resistance, and then impact toughness, density, and thermal conductivity. The best values of the analyzed strength parameters from the point of view of the problem to be solved should be selected as reference points—these can be maximum, minimum, or intermediate values from the experimental sample.

In this case, the method of selection by ordering the objects according to the model is used. In this case, a transition from vector to scalar estimations of objects is necessary. The functions used in solving multicriteria problems play the role of convolution of the vector argument.

To perform scalar optimization, additional knowledge about the properties of generalizing functions, scales of features, and their weight coefficients is needed. Since such knowledge is expert knowledge, the ordering of objects in n-dimensional space cannot be unambiguous. Therefore, it is important to study the influence of the properties of generalization functions and weighting coefficients on the optimization results. The physical and mechanical characteristics of the samples are presented in Table 4.

**Table 4:** Physical and mechanical characteristics of the samples.

No. Samples	Density ρ, g/mm^3^	Bending strength σf, MPa	Compressive strength σc, MPa	Charpy impact strength σi, kJ/m^2^	Thermal conductivity λ, W/(m К)
1	0.00182	7.141	34.328	12.221	0.540
2	0.00161	6.327	32.272	8.255	0.621
3	0.00125	5.554	27.960	8.351	0.800
4	0.00143	5.604	27.881	8.253	0.699
5	0.00151	5.369	27.197	13.587	0.662
6	0.00162	5.482	29.439	27.170	0.617
7	0.00130	4.487	16.765	8.461	0.769
8	0.00129	4.857	19.658	14.298	0.775
9	0.00140	4.236	26.833	8.255	0.714
10	0.00133	4.059	15.091	4.544	0.752
11	0.00140	4.540	17.028	4.566	0.714
12	0.00117	4.513	21.634	3.546	0.893
13	0.00112	3.326	11.131	3.604	0.935
14	0.00112	3.788	16.022	5.525	0.893
15	0.00105	4.254	20.514	4.244	0.952
16	0.00125	3.712	18.173	4.065	0.806
17	0.001197	4.289	20.119	9.070	0.840
18	0.00114	4.668	31.428	6.338	0.877
19	0.00110	3.356	14.377	6.266	0.862
20	0.00120	3.500	15.567	5.628	0.820
21	0.00110	4.183	21.758	3.479	0.877

Relative deviations calculated by the formula (15) *δy*_*ij*_ physical and mechanical characteristics of geopolymers from the optimal value, as well as the values of convolutions (Eqs. 16–18) and criteria (Eqs. 19–21), are presented in Table 4. The calculations were based on the relative change of measured values from their optimal values for the number of samples under study. The maximum and minimum deviation values were calculated for each sample and additive, multiplicative and additional multiplicative convolutions were determined for each sample.

The calculations were performed under the assumption that all criteria have different importance defined above with the help of Markov chains.

For the conclusion regarding the optimal geopolymer composition, it is necessary to take into account the coincidences of different generalization functions and the degree of adequacy of each generalization function to the problem being solved. The analysis of the results presented in Table 5 shows that additive convolution, additional multiplicative convolution, and the Laplace criterion unambiguously point to sample No.6, which corresponds to group FA2 at a fly ash content of 50 g, and the Wald criterion gives close to this result.

**Table 5:** Matrix of dimensionless values of geopolymer parameters, as well as convolution and criteria values.

No. samples	Density ρ	Bending strength σf	Compressive strength σc	Charpy impact strength σi	Thermal conductivity λ	Min	max	ya	yms	ymd
1	1.000	0.018	0.014	0.635	0.012	0.012	1.000	0.415	**0.099**	0.382
2	0.730	0.227	0.101	0.800	0.207	0.101	0.800	0.446	0.342	0.385
3	0.269	0.426	0.285	0.796	0.635	0.269	0.796	0.439	0.402	0.369
4	0.500	0.413	0.288	0.800	0.393	0.288	0.800	0.475	0.453	0.395
5	0.602	0.474	0.317	0.578	0.305	0.305	**0.602**	0.480	0.462	0.401
6	0.743	0.445	0.222	0.011	0.197	**0.011**	0.743	**0.402**	0.241	**0.357**
7	0.333	0.701	0.760	0.792	0.561	0.333	0.792	0.589	0.555	0.467
8	0.320	0.606	0.637	0.548	0.575	0.320	0.637	0.509	0.489	0.416
9	0.461	0.765	0.332	0.800	0.429	0.332	0.800	0.557	0.529	0.450
10	0.372	0.811	0.831	0.955	0.520	0.372	0.955	0.656	0.613	0.507
11	0.461	0.687	0.749	0.954	0.429	0.429	0.954	0.629	0.603	0.491
12	0.167	0.694	0.553	0.997	0.858	0.167	0.997	0.575	0.466	0.459
13	0.103	1.000	1.000	0.994	0.959	0.103	1.000	0.712	0.487	0.542
14	0.103	0.881	0.792	0.914	0.858	0.103	0.914	0.624	0.443	0.492
15	0.013	0.761	0.601	0.968	1.000	0.013	1.000	0.566	0.223	0.457
16	0.269	0.900	0.700	0.975	0.650	0.269	0.975	0.645	0.572	0.502
17	0.201	0.752	0.618	0.766	0.731	0.201	0.766	0.557	0.482	0.448
18	0.128	0.654	0.137	0.880	0.820	0.128	0.880	0.463	0.329	0.390
19	0.077	0.992	0.862	0.883	0.784	0.077	0.992	0.638	0.414	0.502
20	0.205	0.955	0.811	0.910	0.683	0.205	0.955	0.651	0.544	0.507
21	0.077	0.779	0.548	1.000	0.820	0.077	1.000	0.560	0.373	0.453
		Vald	Laplas							
		**0.602**	**0.011**							

Significant values are in bold.

Thus, most of the criterion methods used in this work give practically the same results and allow us to conclude: that the best is the geopolymer composition from the FA2 group with fly ash content of 0.5–0.75 max.

**Figure 3:**
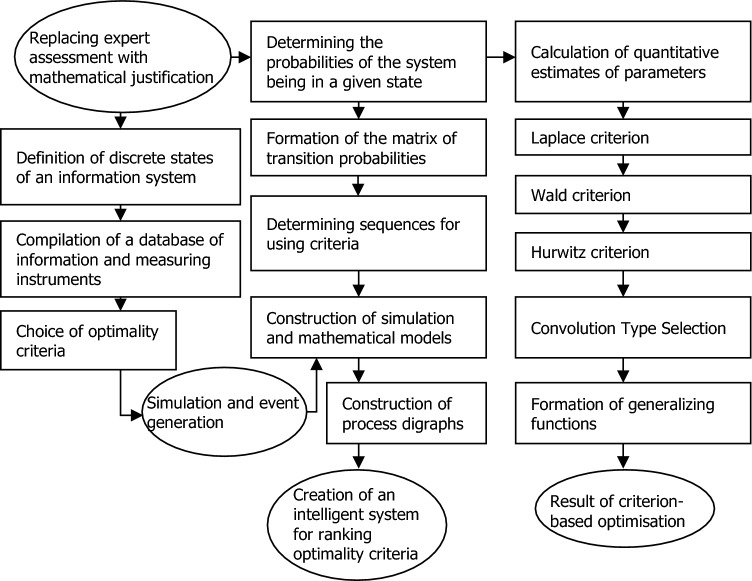
Conceptual model for selecting geopolymer formulations.

The formalization of the main measures to determine the optimal composition of geopolymer matrices based on Markov chains and multicriteria analysis is presented in Fig. 3.

The novelty of the presented conceptual model is the computer support of the geopolymer formulation selection process. The peculiarity of the presented model is that it considers multidirectional criteria in the process of structure formation with a constant share of uncertainty and risk present in this process.

The main stages of determining the optimal components of the geopolymer matrix for noise-protective panels according to the presented conceptual model work with quantitative information, mathematical calculations, storage and exchange of information, and interpretation of results. The sequence of models underlying the methodology includes Markov chains for determining the ranking of criteria, multicriteria optimization models, algorithms, and techniques of their hybrid implementation.

The modeling of changes in the strength properties of geopolymers as parameters of target functions has shown the advantage of evaluating the digitalization of technologies for their production in the analysis of geopolymer properties, where the qualitative side of loosely structured problems is solved using Markov chains, whereas, for quantitative conclusions about the optimal composition of geopolymers, criterion methods are used.

Multicriteria optimization involves the simultaneous optimization of multiple conflicting target functions within a defined domain. When analyzing the impact of ingredient composition changes on the physical properties of geopolymers, the ability to graph these changes becomes increasingly challenging as the number of properties under consideration grows. While graphical representation works for two properties, it evolves into a volumetric representation for three properties. However, beyond this point, with five properties involved, such as density, compression resistance, shear resistance, impact viscosity, and thermal conductivity, visual representation loses its effectiveness. This complexity makes it impossible to identify optimal geopolymer formulations solely through trend analysis.

The multicriteria optimization problem revolves around finding a vector of target variables that adheres to specified constraints, primarily driven by experimental data. In this context, optimization seeks a solution in which the target function values align with the task requirements. It is essential to note that discussing the need for additional experimental studies to validate the proposed model is irrelevant here. This is because the strength and other geopolymer properties obtained in this model do not represent fixed values but rather a representation of the feature space defined by the criteria used. Therefore, the focus lies on ranking these criteria, with the first criterion holding the highest priority in this study.

The integration of modeling, prediction, and optimization in geopolymer formulation replaces expert judgment with mathematical reasoning when selecting diverse geopolymer strength parameters. The presented conceptual model for selectively determining geopolymer formulations through experimental data and their processing via multicriteria optimization methods serves as a methodological tool to enhance the accuracy of geopolymer matrix composition estimations.

## Conclusions

The choice of the optimal formulation of geopolymers is explained by the peculiarities of the methodology used, based on the processing of the available experimental material with discrete changes in properties, the values of which correspond to the data presented in the form of characteristics of the samples under study. When issuing conclusions on the optimization of properties of the used experimental sample, the result is presented in the form of a sample number with properties known in advance.

The use of the multicriteria approach in the presence of several optimality criteria allows us to make adequate decisions on the composition of the geopolymer mixture, which simultaneously provides maximization of the technological and mechanical properties of fly ash used as additives in the composition of geopolymer composites. Increasing the efficiency of such solutions is achieved through the use of hidden opportunities associated with the adaptation of preference selection of factors determining the thermodynamic and strength properties of the mixture and the synthesis of procedures for their selection in the analytical hierarchy of preferences.

Structural compositions and their manifestations can be characterized by independent thermal and physical–mechanical properties, which cover the full range of geopolymer strength manifestations. Markov chains in the system of multicriteria optimization represent a synthetic apparatus accumulating heterogeneous factors of exogenous and endogenous nature. Each state of parameters characterizing the information situation of physical and mechanical property determination at a given formulation of their obtaining is assigned a certain probability of transition matrix. The rows of this matrix contain the states in which the system is currently located, while the columns contain the states to which the Markov chain can transition. This allows us to observe how the Markov chain converges to a stationary distribution and to calculate the weighting coefficients of multicriteria optimization.

Since the task of the research was to select from the whole variety of analyzed experimental data those that correspond to differently directed reference points, maximum values of compressive strength, bending strength, and impact toughness with minimum density and thermal conductivity, which are the means of achieving the goal with the help of the proposed innovative methodology of processing the results, its experimental confirmation is the identification of the available information on the composition and manufacturing technology of geopolymers. Combining subjective and objective elements of selection, and calculations based on the results of experiments, the proposed method of information processing allows for increasing the accuracy of determining the optimum composition of geopolymers that maximize their strength properties.

The points of innovation in the presented methodology are as follows:

- Replacement of expert evaluation in the selection of multidirectional geopolymer strength orientations by mathematical justification;
- construction of a conceptual model of the selective choice of geopolymer formulation using Markov chains and multicriteria optimization;
- rank-based approach to optimization criteria;
- replacement of time references by discretization intervals;
- scalability and adaptation to loadings through scaling;
- scalarization of vector estimates

Multicriteria optimization results for fly-ash as an additive on metakaolin-based geopolymer composites show that the optimal composition of the geopolymer matrix within the selected variation range includes 100 g metakaolin, 90 g potassium activator, 8 g silica fume, 2 g basalt fibers and 50 g fly ash by ratio weight. This ratio gives the best mechanical, thermal, and technological properties.

## Data Availability

All data generated and analyzed during the study are available from the corresponding author upon reasonable request.
